# Comprehensive analysis of Saudi Arabia's research output: a bibliometric study (2013--2022)

**DOI:** 10.12688/f1000research.160450.3

**Published:** 2026-01-28

**Authors:** Bharti Chogtu, Ritheesh V, Ashwath Naik, Santhosh Venkata

**Affiliations:** 1 Kasturba Medical College, Manipal Academy of Higher Education, Manipal, Karnataka, India; 2 Directorate of Research, Manipal Academy of Higher Education, Manipal, Karnataka, India; 3 Manipal Institute of Technology, Manipal Academy of Higher Education, Manipal, Karnataka, India

**Keywords:** Saudi Arabia, Citations, Bibliometrics, Publications, Geography

## Abstract

**Background:**

Bibliometric studies that employ quantitative methods are pivotal for evaluating and analysing the dissemination of scientific knowledge across various disciplines. These studies assess the impact and reach of scholarly works by examining citation patterns, authorship trends, and publication metrics.

**Methods:**

This study examines Saudi Arabia’s research paper output indexed in the Scopus database, utilizing bibliographic data from the SciVal database. SciVal provides comprehensive cross-searching capabilities across various citation indices and databases, offering multidisciplinary information from high-impact journals. The data aggregation from SciVal includes filtering options such as years, subject areas, publication sources, citation counts, and productivity metrics for researchers, institutions, and countries.

**Results:**

This research focuses on Saudi Arabia’s research productivity and the performance of its top universities from 2013–2022. Additionally, information on average per capita GDP and total GDP for the past nine years was sourced from World Bank data. The analysis encompasses all nations that published research productivity articles annually within the specified period. The present study revealed that the research output from Saudi Arabia increased 320%, with an average annual growth rate of 16%.

**Conclusion:**

The present study provides insights into the research productivity of Saudi Arabia and its leading universities, contributing to a broader understanding of global scientific output and its economic context. This study provides an idea to other nations to balance their research output vis-a-vis their economic growth.

## 1. Introduction

Scientific research is a cornerstone of national development, reflecting a country’s intellectual progress and its contribution to global knowledge. In recent years, bibliometric studies have emerged as indispensable tools for understanding the dynamics of research productivity and its broader implications. These studies enable the evaluation of scientific output by examining key parameters such as citation impact, authorship trends, publication metrics, and institutional productivity. These analyses provide evidence-based insights that inform research policy, funding strategies, and academic planning.

Saudi Arabia, the largest country in the Arabian Peninsula, spans approximately 2.15 million square kilometers and is characterized by a harsh, dry desert climate with significant temperature extremes. The population is approximately 36.5 million, predominantly Arab, with a significant proportion of expatriates. ^1^ Academically, Saudi Arabia has made significant strides, particularly under Vision 2030, which aims to diversify the economy and enhance human capital through educational reforms and research initiatives. ^2, 3^ The country has seen an increase in scientific productivity and international collaboration. ^4– 6^ Saudi Arabia has experienced a significant transformation in its higher education sector over the past decade. The Saudi government has significantly increased its spending on education, which includes substantial investments in research and development which has facilitated a rise in research activities and publications, particularly in health sciences and other critical fields. ^7^^,^^8^ Over the past decade, Saudi Arabia has shown a significant transformation in its higher education sector to enhance global research visibility, making it one of the fastest-growing systems worldwide and also actively addressing current challenges faced by government. Saudi universities have adopted strategies for achieving sustainable development goals by adapting the Kingdom of Saudi Arabia’s Vision 2030 framework. ^9^

Several bibliometric studies have been reported in the past indicating the impact of research at the individual, institution, country, etc., levels. A study by Hamdan et al. (2020) ^10^ investigated the relationship between higher education expenditures and economic development in Saudi Arabia over forty years (1978–2017) and reported no significant correlation despite substantial investment in higher education as part of the sustainable development process. Artificial intelligence awareness in higher education at Prince Sattam Bin Abdulaziz University is low, highlighting the need for increased awareness of its educational applications. ^11^ Emirates policies in transnational higher education transform academic institutions globally, impacting students, teachers, and administrators amid shifting globalization dynamics. ^12^ Integrating digital information and tutor quality enhances technology acceptance in education, with user traits and information flow influencing adoption. ^13^ Information communication technology use in Saudi universities is driven by computer self-efficacy, anxiety, and enjoyment, which impact students’ satisfaction and continued use for digital learning. ^14^ King Saud University council members contribute to institutional strategy through role-based responsibilities, technology use, multilevel engagement, and collaborative governance. ^15^

Saudi Arabia has experienced a significant increase in research publications across various fields over the past decade. Saudi Arabia ranks 15th globally in terms of COVID-19 research publications, contributing significantly to the global understanding of the pandemic. ^16^ The integration of information and communication technologies (ICTs) in research practices has been utilized to facilitate knowledge sharing and enhance research performance; as a result, studies have shown that the use of digital tools positively impacts scholarly publications. ^17^ International collaboration has had a constructive effect on the quality of research and visibility of Saudi Arabia in the scientific community. The average annual growth rate of Saudi Arabia’s scientific publications is 17.16%, which is largely driven by international collaborations. ^18^ International collaboration plays a significant role in enhancing the research output of Saudi universities, influencing both the quality and quantity of research publications. Specifically, 64.74% of the country’s scientific output from 2001--2020 was attributed to international collaborations, with the United States and Egypt being the most frequent partners. ^19^ International collaborations facilitate the exploration of diverse research themes and the adoption of advanced technologies. International collaborations are vital for enhancing the research output of Saudi universities, contributing to both the quantity and quality of publications. There is a growing cultural emphasis on education and the dissemination of knowledge in Saudi Arabia. This cultural shift is supported by institutional policies that promote a collaborative and innovative research environment. ^20^ Government policies play a significant role in the growth of research publications in Saudi Arabia. The Saudi Amended Incentive Program, introduced in 2008, significantly increased research production, leading to a sevenfold increase in published papers between 2009 and 2019. ^21^ Saudi Arabia’s transition from an oil-dependent economy to a knowledge-based economy has been supported by government policies that include significant investments in higher education and research infrastructure. ^22^^,^^23^ Government policies in Saudi Arabia have been instrumental in fostering the growth of research publications by providing financial incentives, establishing research centers, and promoting international collaboration.

Organizational, individual, and technology factors drive creativity in Saudi higher education institutions (HEIs), fostering research and teaching innovation for a knowledge-based economy. ^24^ Research infrastructure, knowledge generation, and organizational support drive the research culture in Saudi higher education institutions, enhancing faculty performance but not mitigating unproductive behaviours. ^25^^,^^26^ Qualitative researchers in the Kingdom of Saudi Arabia (KSA) face challenges related to organizational, social, cultural, and methodological factors, highlighting the need for institutional support and policy reform. ^27^ High-performance HR practices such as training, recognition, and internal mobility increase faculty career success and research performance, whereas decision-making participation hinders research output. ^28^ Saudi technology and innovation (T&I) research grew significantly from 1990–2019, with a surge in publications after 2010, driven by pedagogy-focused studies and expanding university translation departments. ^29^ A 44-item scale with 5 dimensions was validated to measure perceived IT service quality in higher education, which was confirmed through confirmatory factor analysis. ^30^

Open Educational Resources (OER) adoption in higher education is influenced by relative advantage, observability, complexity, and compatibility, with calls for initiatives to address trialability and compatibility challenges. ^31^ The KSA ranks 41st globally in open access (OA) publications, with significant growth in the past decade, led by King Saud University, and the most cited works are from King Abdulaziz University. ^32^ Medical students at Majmaah University relied on online resources during the COVID-19 pandemic, with males and high-GPA students shifting more, highlighting the need for proactive academic support. ^33^ Global university OA adoption is tracked for 963 institutions, highlighting methodological challenges and the need for better open science (OS) policy indicators. ^34^ COVID-19 research highlights China’s leadership and growing global collaboration, with 91.4% of 2020 publications providing open access, reflecting urgent efforts to share knowledge. ^35^ Library and information science (LIS) research in the Arab region has grown significantly, with Kuwait and Saudi Arabia leading, with a focus on academic libraries, bibliometrics, and social media, whereas areas such as artificial intelligence (AI) and digital libraries need more attention. ^36^

In conclusion, Saudi Arabia’s research landscape is witnessing a paradigm shift driven by strategic investments, targeted research domains, comprehensive funding mechanisms, and the integration of advanced technologies. The findings serve as benchmarks for policymakers, researchers, and academic institutions to identify opportunities for enhancing research impact and aligning scientific endeavors with national development goals. Additionally, to contextualize these findings, the study incorporates economic indicators such as average per capita gross domestic product (GDP) (current US$) and total gross domestic product (GDP) (current US$) for Saudi Arabia during the same period, derived from World Bank sources. In addition, we hope to provide national benchmarking data that can be helpful to researchers for the purposes of planning and monitoring institutional development for the purposes of performance review. The objective of the present study is to provide an updated and systematic examination of the development and current state of research productivity in Saudi Arabia.

### 1.1 Details of the data

Bibliometric review involves quantitative methods to analyze the Saudi Arabia’s research, exploring publication history, trends in publication keywords, and citations. The methodology applied in the present study was bibliometric analysis, and we focused on evaluating published papers provided by the SciVal tool via the Scopus database. In the present research, the authors conducted a bibliometric analysis to evaluate published paper in Saudi Arabia’s over an ten year periods, i.e., 2013-2022. The author team searched the SciVal database ( http://www.scival.com) from which publications were retrieved, as SciVal is an analytics platform powered by the Scopus database and was selected for its comprehensive citation metadata, standardized subject classification, and reliable bibliometric indicators widely used in research evaluation. In this study, publications were retrieved using the country-level classification, where Saudi Arabia was selected as the country entity. The primary search limiting the search to keywords and publications from 2013 to 2022. Only original articles from this period were included, while editorials, conference papers, reviews, and other types of publications were excluded. Publications in all languages were considered, with no additional filters applied.

Preliminary analyses were conducted using SciVal tools to gather information about authors, affiliations, and journals. The retrieved data were downloaded from SciVal, and the metadata of the final collection were exported to a CSV file. Statistical analyses were performed using SPSS 20.0 (IBM SPSS Statistics). Continuous variables were described using means ± standard deviations, and categorical variables were presented as percentages. Pearson’s chi-square test or Fisher’s exact test was used to compare categorical variables, while an independent sample t-test was used to analyze mean differences between two groups.

Spearman’s rank correlation coefficient was calculated to assess correlations between variables, with values ranging from -1 to +1. The rho score interpretation was as follows: <0.25 indicated a weak correlation, 0.25–0.49 a moderate correlation, 0.50–0.74 a strong correlation, and >0.75 a robust correlation. A p-value of 0.05 was adopted as the significance threshold.

## 2. Methods

This is bibliometric analyses. It focused on evaluating the publications on Saudi Arabia’s original research paper output indexed in the Scopus database. There are prominent databases available for conducting certain types of analysis, including bibliometric analysis. Scopus database is chosen because it considered as largest citation and abstract database of peer-reviewed literature which covers a wide array of subjects. ^37^ Data were extracted from the Scopus database of Saudi Arabia’s research productivity as well as the productivity of these nations’ top universities between the period of 2013 to 2022 as provided in the SciVal database. The bibliographic data were retrieved from the SciVal database, a Web-based database that provides simultaneous cross-searching of a range of citation indices and databases, with multidisciplinary information from high-impact journals. It aggregates bibliographic data from the SciVal citation indices and offers various data filtering options, such as years, subject areas, sources of publication, citation counts, productivity of researchers, institutions, countries, etc. In this study, we also gathered the information regarding their average per capita GDP and total GDP from World Bank sources for the previous nine years. The analysis included all the nations that published research productivity articles annually between 2013 and 2022. Bibliometric studies have become an essential tool for evaluating and analysing the proliferation of scientific knowledge within various disciplines. By employing quantitative methods, these studies assess the impact and reach of scholarly works through the examination of citation patterns, authorship trends, and publication metrics.

### 2.1 Publications

To identify the total Saudi Arabia’s research output, a country field tag, Saudi Arabia, was used in the SciVal database as a search option. Publications published from 2013–2022 were considered for the analysis in the proposed work. With this method, a total of 296599 papers were retrieved. The retrieved documents were then refined by using the “publication type” section to identify Saudi Arabia’s research output in journal articles. Only journal articles were included in this study, and other documents, such as conference papers, letters, reviews, editorials, and meeting abstracts, were excluded. For this study, we included 238366 articles, and the publication years of the included articles are presented as both numbers and percentages. The number of publications increased significantly over the study period, rising from 11,695 articles in 2013 to 49,136 articles in 2022 , representing an overall growth of approximately 320% ( Table 1). Publications increased consistently between 2013 and 2022, with a significant acceleration in the last few years. The change in pattern suggests that academic activity and research output increased significantly throughout this time. A productivity index was calculated to compare the individual growth trend according to its baseline in 2013, defined as (Current Year Total – 2013 total)/2013 Total. ^45^

**Table 1.** Yearwise growth of publications.

Year	Publications (%)	Growth rate	Average growth rate (%)
2013	11695 (4.9%)	0	0
2014	14232 (6.0%)	2537	21.7
2015	15921 (6.7%)	1689	11.9
2016	16979 (7.1%)	1058	6.6
2017	17232 (7.2%)	253	1.5
2018	18774 (7.9%)	1542	8.9
2019	22497 (9.4%)	3723	19.8
2020	31436 (13.2%)	8939	39.7
2021	40464 (17.0%)	9028	28.7
2022	49136 (20.6%)	8672	21.4
Total	238366	37441	Average = 16.0

^#^ Average growth rate (%) was calculated to compare the individual growth trend according to its baseline in 2013, defined as (number of publications in the present year – number of publications in the base year)/number of publications in the base year.

Table 1 reflects the yearly distribution of articles published in the journal within the specified period (2013–2022) and shows the percentage of the number of articles published each year. The figure also revealed that a total of 238,366 research papers were published during this period, with the average annual growth rate over the period was 16.0%. There is an increase in the number of publications found each year, and it continued in all the later years of study. The highest growth rate was recorded in 2020, with a growth rate of 39.7%, followed by 2021, with a growth rate of 28.27%. The growth rate is calculated via the following formula. It is evident from the figure shown above that the number of articles published each year in this journal tends to be used to measure the growth of the literature.

**Figure 1.**
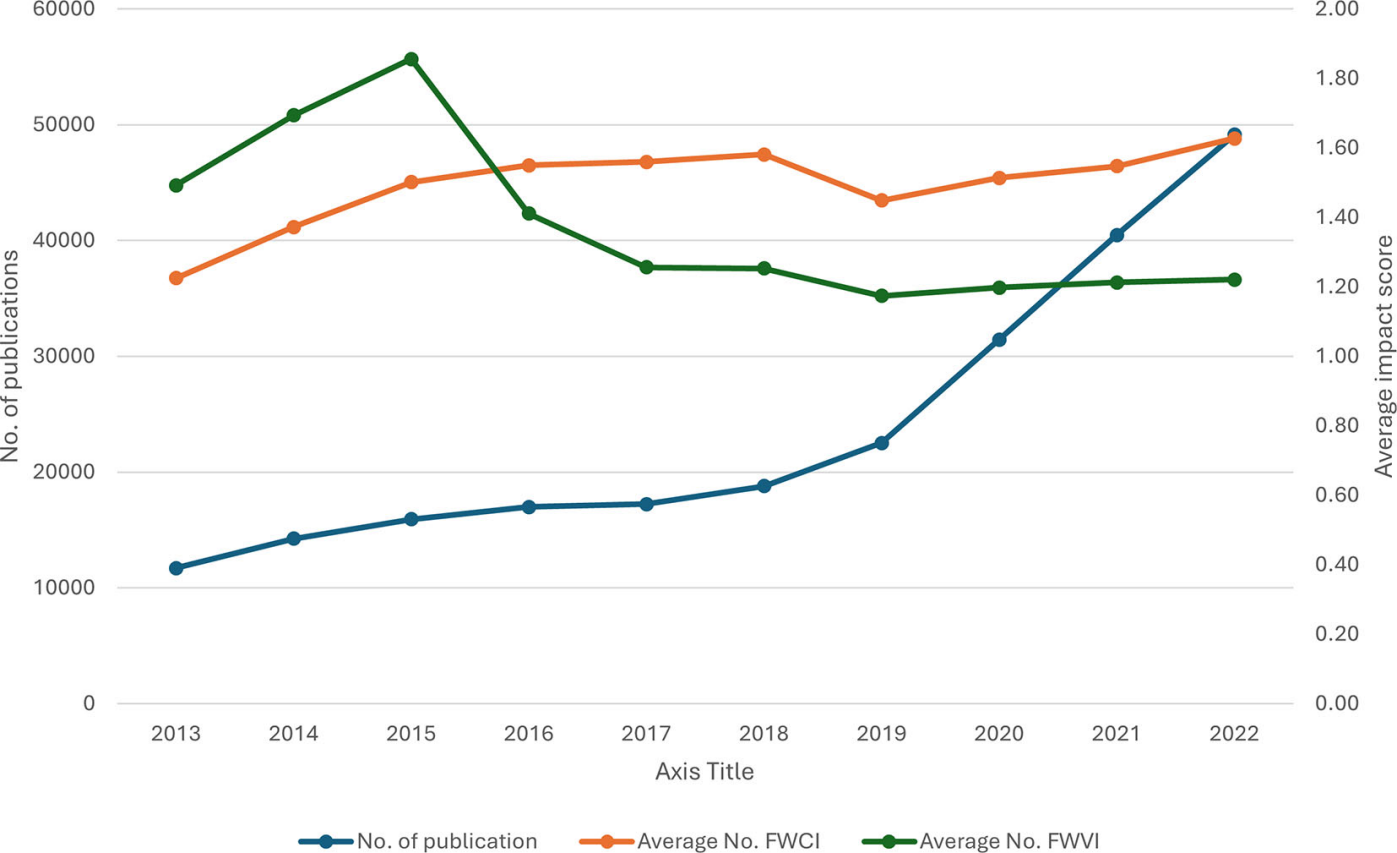
Publication trends with the FWVI and FWCI over time.

Figure 1 depicts the yearly total number of publications and the average impact score of the field-weighted view impact (FWVI) and field-weighted citation impact (FWCI) received per paper per year. Importantly, there has been a substantial and consistent increase in the number of publications over the years, and the FWCI shows a general upwards trend, indicating improved citation impact over time; however, despite the increasing number of publications, the relative viewership impact has diminished over the years. The continuous increase in the number of publications is accompanied by a steady rise in the FWCI, with a significant positive correlation (R ^2^ = 0.66, P = 0.039) across the years. This finding indicates that the increase in volume has not diluted citation quality; rather, it has improved it. In contrast to the FWCI, the FWVI is not positively correlated (R ^2^ = -0.83, P = 0.003) with an increase in the number of publications. Compared with those of the FWCI and FWVI, both metrics initially improved, but after 2015, their paths diverged. The FWCI continued to rise, indicating increasing citation impact, whereas the FWVI declined.

**Figure 2.**
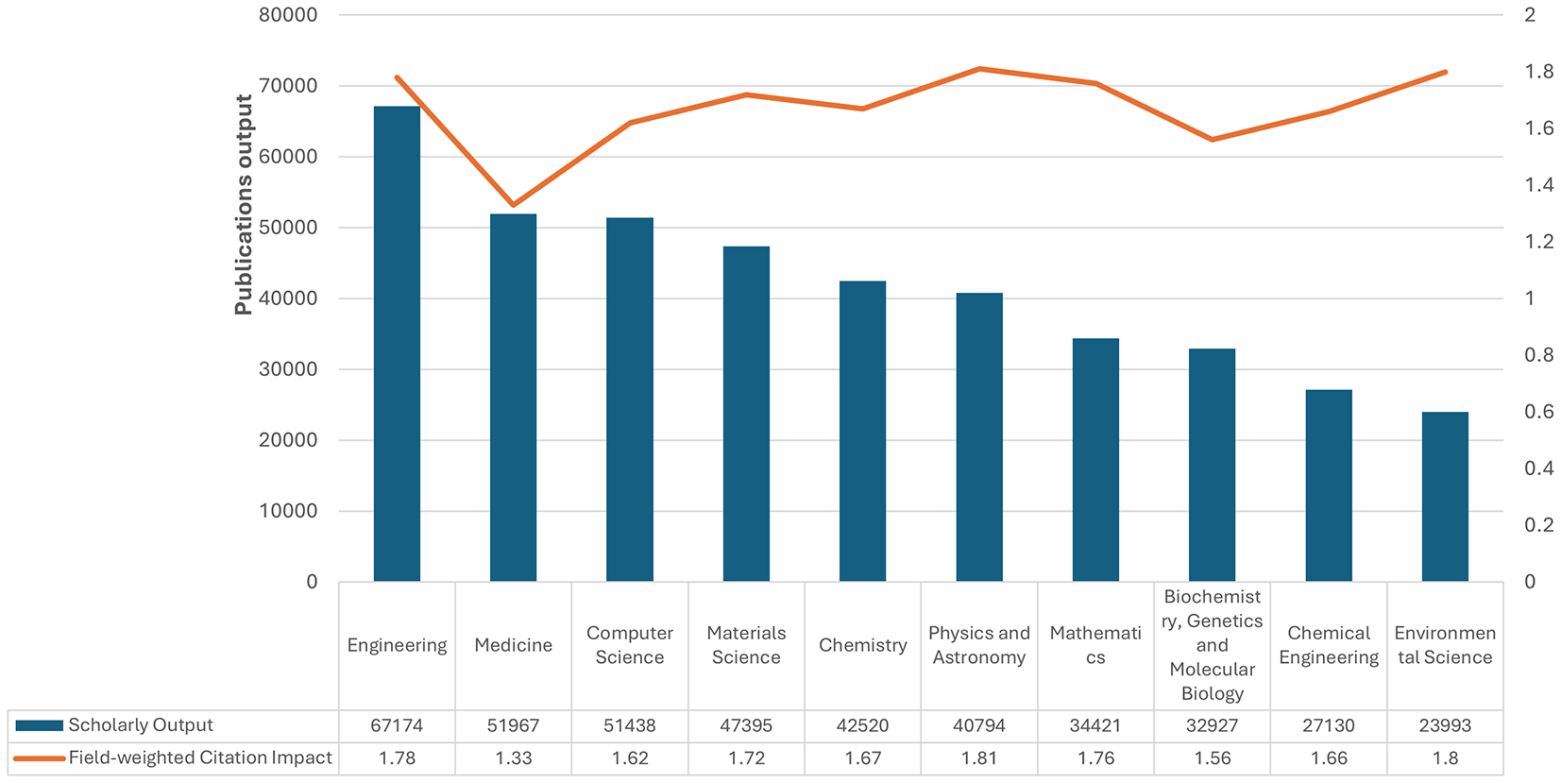
Scholarly output and field-weighted citation impact across disciplines in Saudi Arabia.

Figure 2 Saudi Arabia’s scholarly output and field-weighted citation impact, 2013–2022: There is clearly substantial variation across research productivity and impact. While leading in the volume of scholarly outputs at 67,174 publications, the field-weighted citation impact was almost lower at 1.78 than that of other disciplines. Medicine followed with 51,967 and a citation impact of 1.33. In the fields of computer science and materials science, large outputs are found, with 51,438 and 47,395 publications, respectively, with higher citation impacts of 1.62 and 1.81, respectively, and thus more substantial influence per publication. Chemistry, Physics, Astronomy, and Mathematics also have well-balanced contributions, with outputs of 42,520–34,421 and citation impacts of approximately 1.67–1.76. Although lower outputs can be found in biochemistry, genetics, and molecular biology, with 32,927, their impact of 1.56 is remarkable. Another example includes Chemical Engineering and Environmental Science, with outputs of 27,130 and 23,993, respectively, which amount to robust citation impacts of 1.66 and 1.80. These data demonstrate the diverse research landscape in Saudi Arabia, which manifests high productivity and considerable implications across many fields of science.

**Figure 3.**
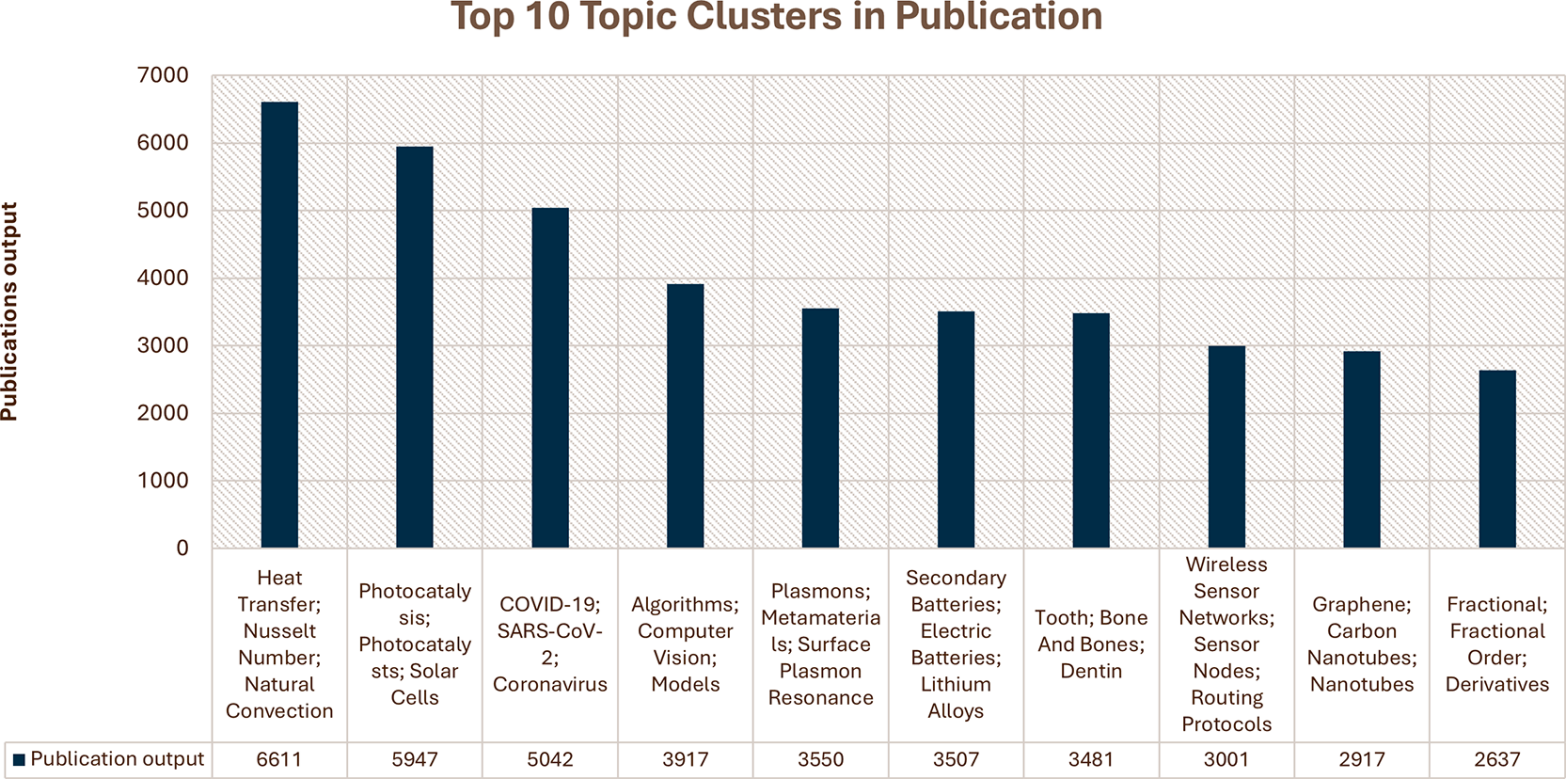
Top 10 research topic clusters in Saudi Arabian publications.

As shown in Figure 3, the top 10 research topics in Saudi Arabian publications for the period from 2013–2022 range from a wide array of focus areas, thereby underpinning the breadth of research initiatives the country has undertaken. For heat transfer, the Nusselt number is the most prolific cluster, with nearly 7,000 publications, underlining a strong orientation toward thermodynamics and related engineering applications. The next ones are “Photocatalysis” and “COVID-19; SARS-CoV-2,” with over 5,000 publications each, reflecting global and regional priorities in renewable energy solutions and pandemic-related research. Other key clusters are “Algorithms, Computer Science,” “Plasmons, Metamaterials,” and “Secondary Batteries,” each contributing 3,000–5,000 publications on improvements in computational techniques, nanotechnology, and energy storage technologies. Intense research has also been conducted in “Tooth; Bone and Bones,” “Wireless Sensor Networks,” “Graphene; Carbon Nanotubes,” and “Fractional; Fractional Calculus,” all with large outputs, which thus focus on biomedical engineering and communication technologies, as well as advanced materials science. This broad list of productive research areas highlights the comprehensive and multidisciplinary approach Saudi Arabia has toward scientific inquiry.

**Figure 4.**
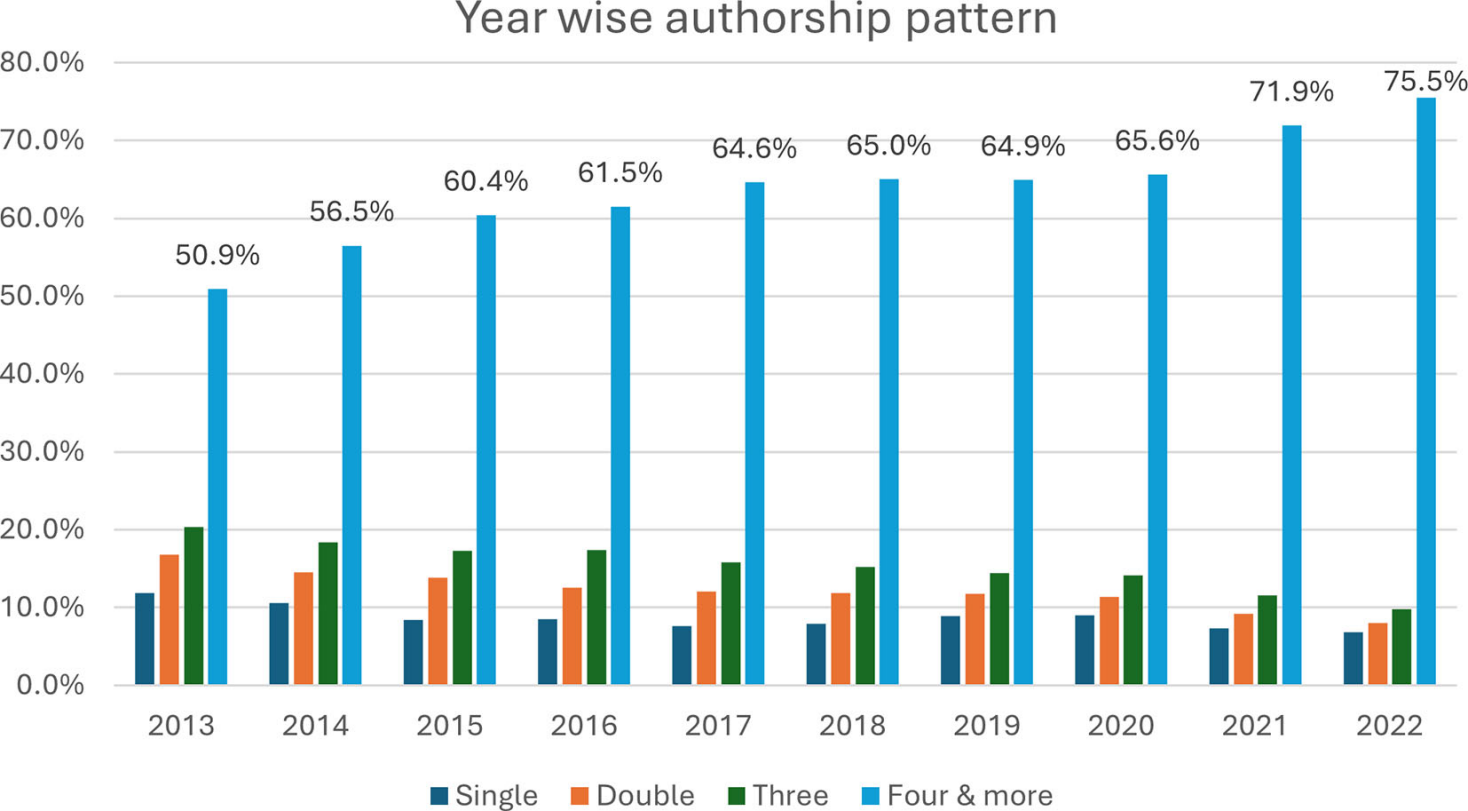
Yearly trend in the number of authors per publication.

Figure 4 represents a comprehensive view of authorship trends in academic publications over a decade, from 2013–2022. The number of authors per publication is based on the figure provided, and we can observe trends and changes in authorship over the years. A Kruskal–Wallis H test was used to compare the median number of authorships per publication across the years, resulting in a p value of 0.001. The data highlight that the median number of authors per article increased from four (IQR: 1–2263) in 2013 to six (IQR: 1–2302) in 2016. This highlights that the median number of authors per publication has shown a clear upwards trend and that the number of authors per paper has significantly increased over the 10-year period (P <0.001). The data from 2013–2022 show a clear pattern of decreasing isolated events (singles and doubles) and a corresponding increase in larger clusters (three occurrences and four or more occurrences). In 2013, publications with four or more authors constituted 50.9% of the total, and this proportion grew to 75.5% by 2022. In 2013, single-author publications made up 11.9% of the total, but by 2022, this figure had dropped to just 6.8%. This decline reflects a growing trend towards collaborative research.

**Figure 5.**
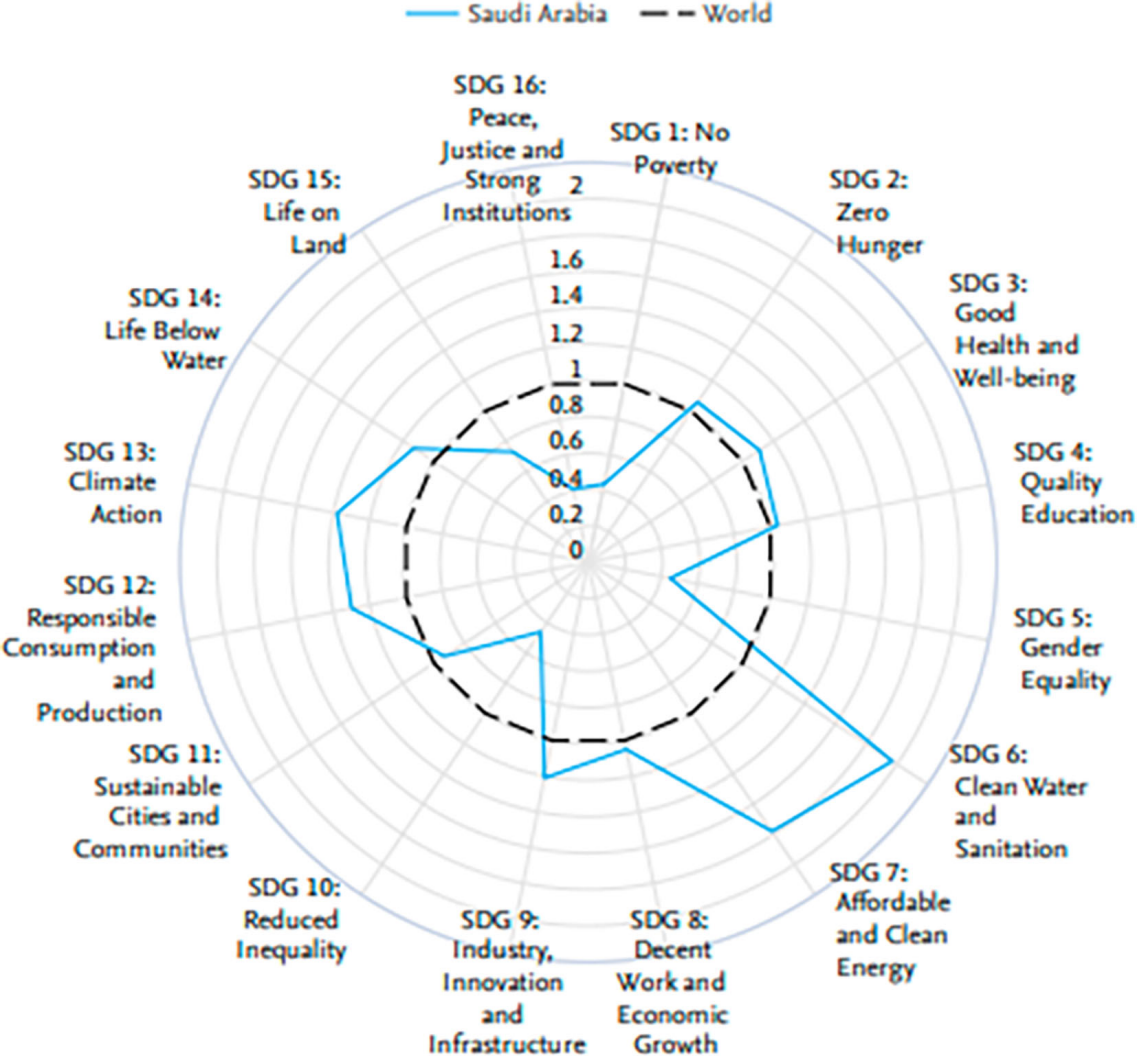
Comparison of the performance of Saudi Arabia in terms of UN sustainable development goals (SDGs).

The radar chart in Figure 5 reveals Saudi Arabia’s excellent performance on some of the United Nations Sustainable Development Goals, 2013–2022, against the world average. Saudi Arabia is exemplary in SDG 7, Affordable and Clean Energy, and SDG 9, Industry, Innovation, and Infrastructure, above the world average. This redundantly underlines that this nation is rightly committed to developing clean energy agendas and infrastructural development. Moreover, Saudi Arabia performs well with respect to SDG 16 in terms of peace, justice, and strong institutions and with respect to SDG 8 in terms of decent work and economic growth, hence truly succeeding in building a society that works both stably and moderately and is economically dynamic. This means that the country does show its commitment to sustainable development and what it has been able to do thus far in taking concrete initiatives toward pursuing some of the critical global challenges that affect humankind.

## 3. Analysis

**Figure 6.**
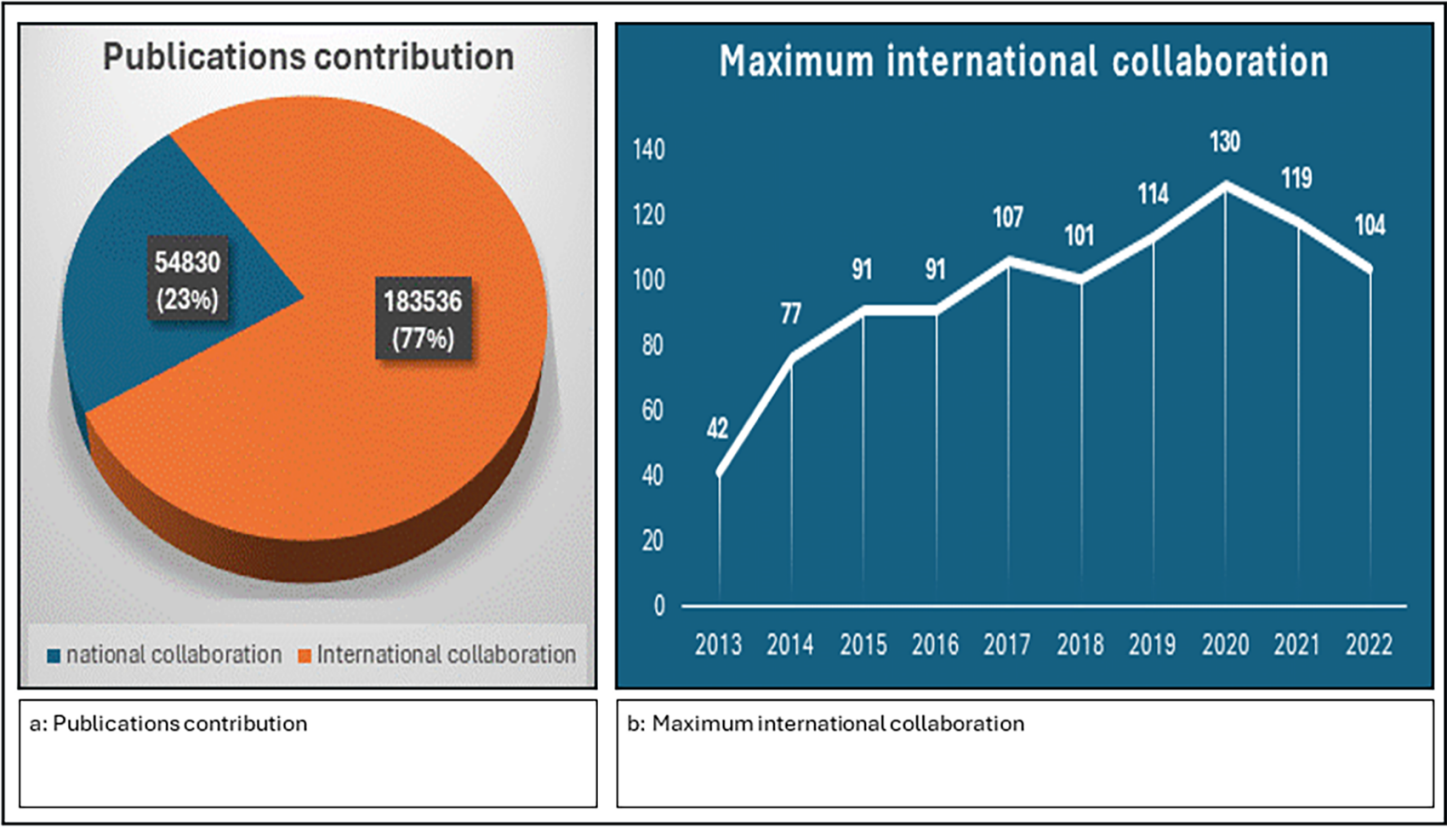
Global collaboration in scientific publications: Yearly trends in international partnerships.

The data on publications from national and international collaborations paint a vivid picture of the evolving landscape of scientific research. A higher count of publications suggests international collaboration and greater visibility, knowledge exchange, and potential for broader societal impact. International collaborations clearly dominate in terms of sheer volume, reflecting the global nature of modern scientific inquiry and the increasing emphasis on cross-border collaboration, as shown in figure international collaboration accounts for a substantial majority of the output (183,536 publications; 77%), while nationally collaborative publications constitute a smaller proportion (54,830 publications; 23%) ( Figure 6a). The data provided the maximum number of international collaborations for each year from 2013–2022, as shown in Figure 6b. The maximum number of international collaborations varied each year, ranging from 42 in 2013 to 130 in 2020. There are fluctuations in the maximum number of international collaborations across the years, with some years experiencing significant increases or decreases compared with the previous year, with 2020 standing out as the year with the highest maximum international collaborations, reaching 130.

**Figure 7.**
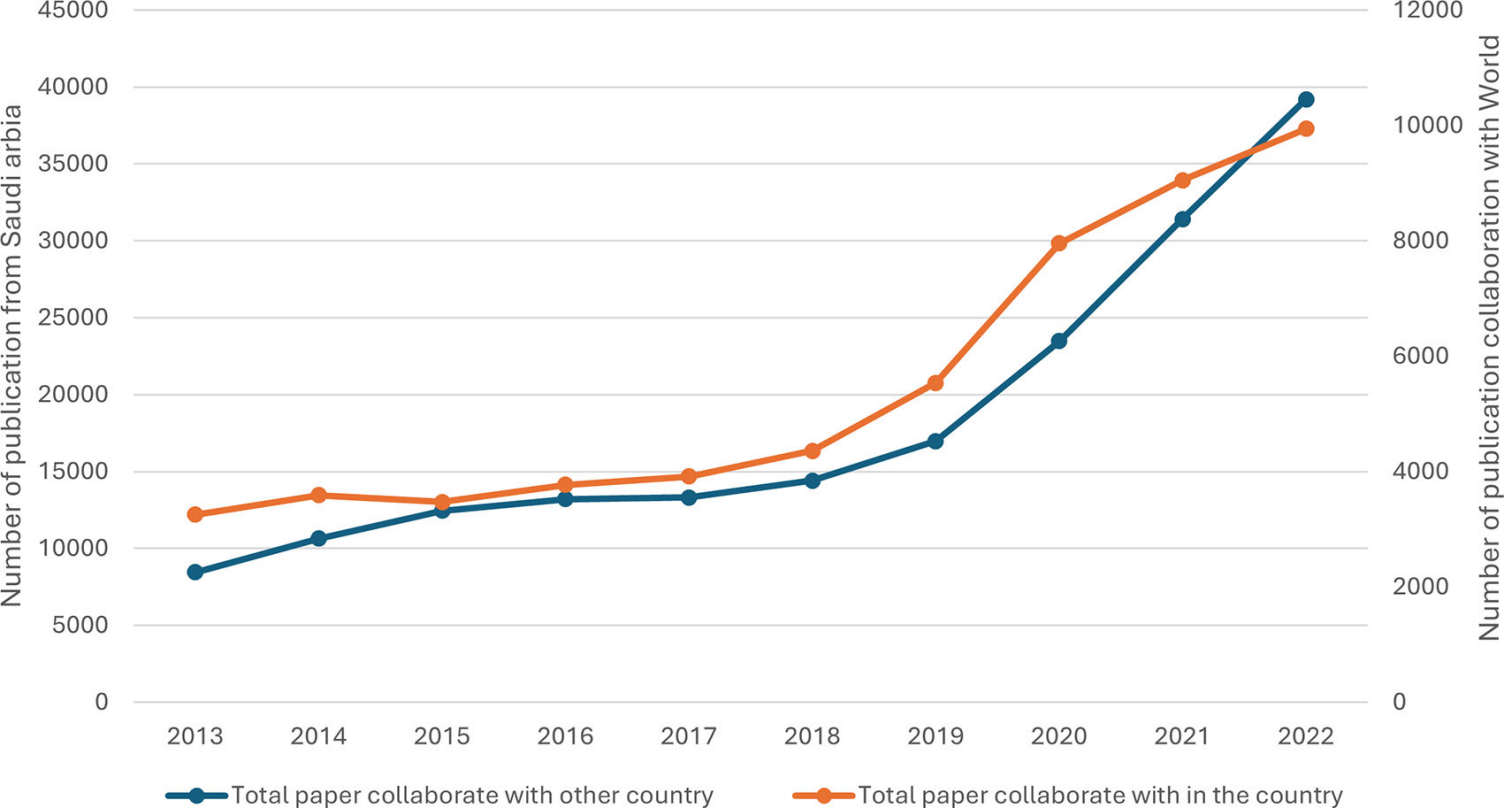
Trends in national and international research collaborations.

Figure 7 shows that both national and international collaborative papers consistently and significantly increased over the observed period. This indicates a growing trend towards collaborative research, both within the country and internationally. The number of papers resulting from international collaboration increased more than fourfold from 2013 to 2022. There was a noticeable acceleration in growth from 2018 onwards, and the highest growth occurred between 2019 and 2020, where the number of papers surged by 6,520, reaching 23,483 in 2020, indicating a significant 38.4% increase. The number of national collaborative papers increased steadily from 3,255 in 2013 to 9,944 in 2022. The average annual growth rate for national collaborations stands at approximately 16.9%, reflecting a steady but slightly slower expansion than that of international collaborations. While both types of collaboration have increased significantly, international collaboration has seen a more substantial numerical increase than national collaboration has, but national collaboration has shown consistent proportional growth, indicating a sustained effort within institute collaborative research. In conclusion, a positive correlation of r = 0.988, p value = 0.001 between national and international publications in Saudi Arabia indicates a very strong relationship, suggesting that efforts to promote research effectively contribute to both national and international scholarly outputs.

From Table 2, a Independent sample t-test was conducted to compare the mean FWVI and FWCI scores between country-by-country collaborative and national collaborative publications, resulting in a p value of 0.001. This indicates a statistically significant difference between the mean FWVI and FWCI scores in publications between country-by-country collaborative and national collaborative publications. The data highlight the potential impact of collaboration in publications on citation metrics, with multiple collaboration papers (1.41 ± 3.47) showing a higher mean FWVI in publications but also greater variability than national collaboration papers (0.98 ± 1.34), similar to FWCI citations. Overall, the data suggest that international collaborations have a more substantial impact in terms of both views and citations than do national collaborations.

**Table 2.** Differences in impact scores between international and national collaboration research outputs.

	National collaborations	International collaborations	Result
	Mean ± SD	Mean ± SD	P value *
FWVI	0.98 ± 1.34	1.41 ± 3.47	<0.001
FWCI	0.84 ± 1.48	1.73 ± 6.68	<0.001

* Independent sample t-test.

**Figure 8.**
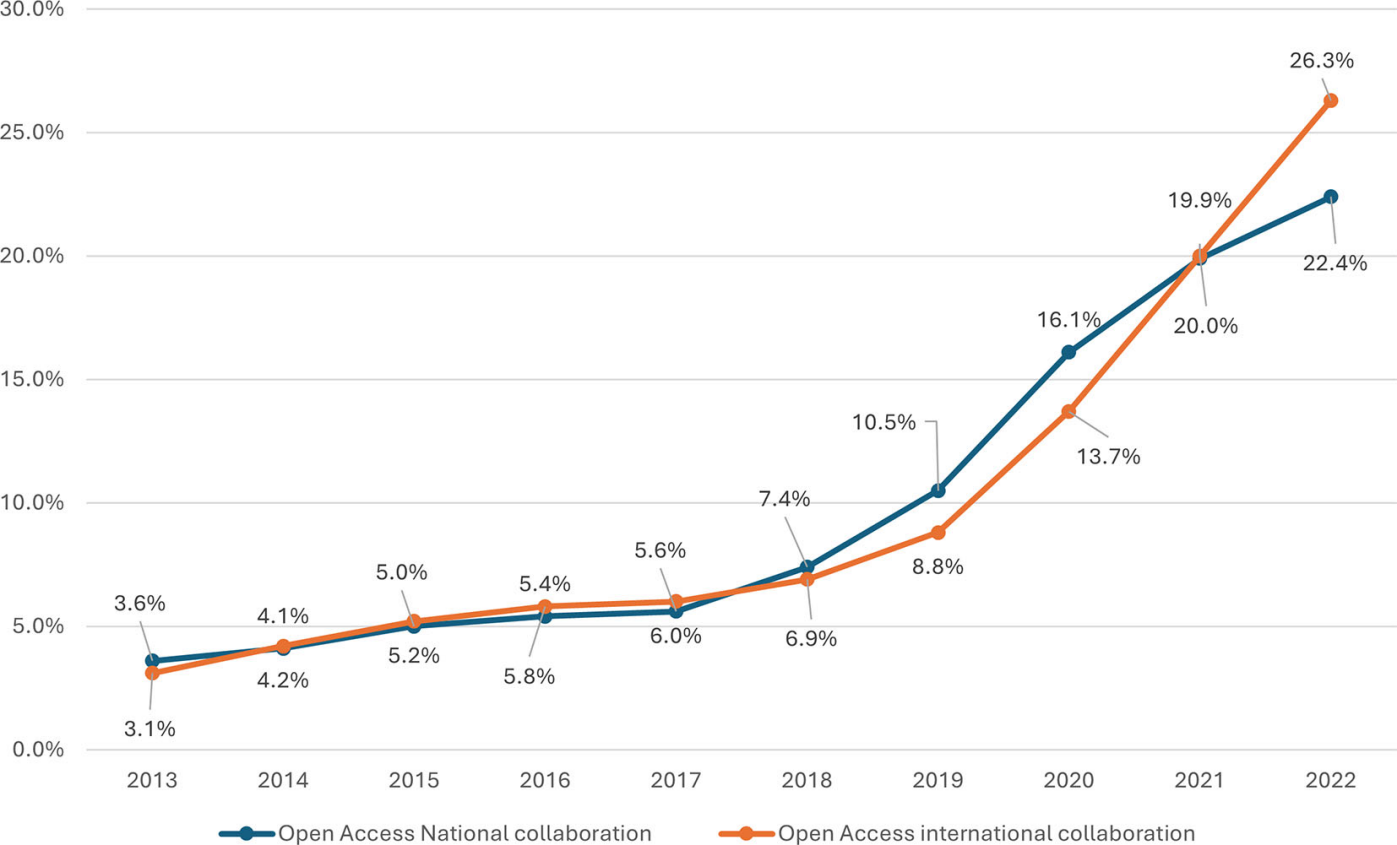
Growth of OA publications with and without international collaboration.

Both national and international open access collaboration have shown a consistent upwards trend over the observed period, as shown in Figure 8. The percentage of open access publications from national collaborations started at 3.6% in 2013 and increased steadily each year, reaching 22.4% by 2022. This growth is notable, showing a more than sixfold increase over the decade. Significant annual increases are observed, particularly from 2018 onwards, where the percentage jumped from 7.4% to 10.5% in 2019, marking a substantial 3.1% increase in a single year. The highest growth rate was observed between 2019 and 2020, with a 5.6% increase. International collaboration in open access started slightly lower than national collaboration at 3.1% in 2013 but experienced a more dramatic rise, reaching 26.3% by 2022. This represents an eightfold increase. The data reveal a steady increase each year, with notable jumps between 2019 and 2020 (from 8.8% to 13.7%) and a significant increase from 2021 to 2022 (from 20.0% to 26.3%). These jumps suggest potential influences on increasing global research networks. In conclusion, a positive correlation of r = 0.979, p value = 0.001 between national and international open access publications in Saudi Arabia indicates a powerful relationship, suggesting that efforts to promote research in open access publications effectively contribute to both national and international scholarly outputs.

In assessing the patterns of collaboration in scientific research by Saudi Arabia, researchers with others in the rest of the world were included. The top 10 foreign organizations collaborating with Saudi Arabia organizations in research contributed 183536 papers. Egypt leads with a substantial 17.0% of the total 183,536 collaborative documents, highlighting strong research collaboration. India (5.8%, 10560 papers) and the United States (5.0%, 9115 papers) follow closely, demonstrating their role in producing impactful research with Saudi Arabia, followed distantly by Pakistan (5.0%, 9112 papers), China (2.9%, 5279 papers), Tunisia (2.0%, 3702 papers), the United Kingdom (2.0%, 3603), Malaysia (1.7%, 3030 papers), Canada (1.6%, 2854 papers), and Australia (1.0%, 1767 papers) from 2013–2022, as shown in Table 3. Comparative analysis of scholarly publications and their citation impact for Saudi Arabia and its collaborating countries, 2013–2022. Malaysia achieves the highest average FWVI at 1.61, suggesting that its collaborative publications with Saudi Arabia receive significantly more attention than the world average does, and Australia receives notable attention for their research, as indicated by their high FWVI score of 1.46, indicating that their collaborative work is well recognized and viewed. Similarly, China has the highest FWCI at 2.53, indicating that its collaborative research with Saudi Arabia is exceptionally impactful. However, at least from the data presented, what was surprising was that too many such publications were not matched by a proportionate high citation impact or other such measures. Some collaborating countries show a reduced count of publications offset with an impact score, hence a focus on creating impactful research. It also exemplifies how enhancing the quality and impact of research is as important as increasing scholarly output to achieve greater recognition and influence in the eyes of the global academic community.

**Table 3.** Top ten countries that collaborated in publications with Saudi Arabia, 2013–2022.

COUNTRY	Documents (n=183536) (%)	Average FWVI	Average FWCI
Egypt	31250 (17.0%)	0.97	1.08
India	10560 (5.8%)	0.96	1.26
United States	9115 (5.0%)	1.34	1.44
Pakistan	9112 (5.0%)	0.87	1.45
China	5279 (2.9%)	1.29	2.53
Tunisia	3702 (2.0%)	0.92	0.86
United Kingdom	3603 (2.0%)	1.27	1.23
Malaysia	3030 (1.7%)	1.61	1.17
Canada	2854 (1.6%)	1.11	1.05
Australia	1767 (1.0%)	1.46	1.33

From Table 4, we observe that Saudi Arabia has made significant strides in higher education and research, reflected in the performance of its top universities in the Times Higher Education (THE) rankings. This report provides a detailed analysis of the performance of the leading Saudi Arabian universities on the basis of various metrics. The University King Saud University is ranked 401–500. It has the highest number of total publications among the universities listed, with 49,288 publications. Prince Sultan University (PSU), which is also in the 401–500 range, excels in international collaboration (83.6%) and open access publications (64.2%), indicating a strong global research presence. Furthermore, PSU’s field-weighted view impact of 1.42 and field-weighted citation impact of 2.09 are the highest among the listed universities, reflecting its research’s substantial influence and visibility. In summary, while King Fahd University of Petroleum and Minerals is top ranked, Prince Sultan University stands out for its high research impact and global collaboration. King Saud University leads in research output, demonstrating significant productivity. Each institution has unique strengths, contributing to a diverse and dynamic research landscape in the region.

**Table 4.** Research productivity ranking of Saudi Arabia’s top five universities.

Sl. No.	Name of University	The ranking	Total publications	International collaboration paper	Open access publication	FWVI (Mean & SD)	FWCI (Mean & SD)
1	King Fahd University of Petroleum and Minerals	201-250	13165	8117 (61.7%)	4231 (32.1%)	1.24 ± 1.16	1.48 ± 2.24
2	King Abdulaziz University	251-300	44849	37282 (83.1%)	21097 (47.0%)	1.27 ± 2.13	1.90 ± 9.84
3	King Saud University	401-500	49288	37055 (75.2%)	25479 (51.7%)	1.23 ± 3.67	1.46 ± 4.52
4	Prince Sultan University (PSU)	401-500	3440	2874 (83.6%)	2210 (64.2%)	1.42 ± 1.41	2.09 ± 3.28
5	Al-Imam Muhammad Ibn Saud Islamic University	401-500	3827	2835 (74.1%)	1897 (49.6%)	1.05 ± 1.51	1.21 ± 2.60

This reflects high-level results on the number of publications published, their citation impact and how well research institutions internationally collaborate in science. This underscores the country’s strong emphasis on research quality and quantity over the years, with the year-to-year rise in publishing volume accompanying the increasing citation impact. Second, the table highlights Saudi Arabia’s success in working in partnership with universities and research institutions worldwide to build a broader and more international research base. The total number of publications included in this study was 238366. The mean per capita GDP for Saudi Arbia countries is 797.3±130.8 US$ (per billion dollars). The Pearson correlation coefficient between the GDP per capita and total number of research documents among Saudi Arabia countries during the period 2013–2022. Analysis revealed a moderate to strong positive association between per capita GDP and research outcomes (r = 0.600, p value = 0.067). However, this relationship did not find a statistical significance (p value = 0.067).

Moreover, from the table, we can observe that there are strong positive (low correlation values) relationships between the number of publications as well as the citation impact and international collaboration. These results infer that enhanced collaborative efforts and greater publication productivity are correlated with better citation impact, thus verifying the overall strategy of the Kingdom of Saudi Arabia towards collaboration and abundant research publishing culture to ensure an improvement in quality as well as in conducting research with global outreach. It is a trajectory that aligns with Saudi Arabia’s ambition to improve its academic and scientific rank on the global map.

## 4. Discussion

This study comprehensively examines Saudi Arabia’s evolving research landscape from 2013 to 2022, highlighting the nation’s strategic investments in higher education and research infrastructure. During this period, the country’s research output surged by an unprecedented 320%, with an average annual growth rate of 16%, demonstrating its commitment to strengthening its global research presence. Notably, a substantial proportion of this output is concentrated in health sciences and medicine, with institutions focusing high-impact domains such as clinical care, medical procedures, and pharmaceutical sciences. ^7^^,^^38^ This sustained growth trajectory underscores the nation’s deliberate efforts to become a significant contributor to the international research community.

The thematic trajectory of Saudi Arabia’s research reveals a shift toward diverse, interdisciplinary, and high-impact domains. Academic institutions such as King Saud University and Prince Sultan University have played instrumental roles in this transformation. It contributed extensively to research with a high volume of publications. Institutions like King Saud University success attributed to visionary approach, substantial funding, and faculty contributions ^39^ and Prince Sultan University is a leading private university in Riyadh by categorizing programs into architecture-related, technology-related, management-related, and traditional engineering programs. ^40^ These universities have demonstrated exemplary performance, particularly in fostering robust international collaborations and producing high-impact research outputs. The study highlights a significant fourfold increase in international collaborations, particularly with countries such as Egypt, India, and the United States. Co-authored publications with international first authors achieve a higher rate of citations than publications with domestic first authors, indicating the constructive effect of international collaborations on the quality of research and perceptibility of Saudi Arabia in the scientific community. ^19^ The average growth rate of Saudi Arabia’s scientific publications is attributed more to international collaborations than domestic collaborations. It involves a significant percentage of international collaborations, with the United States and Egypt being the highest collaborating countries ^19^ establishing robust collaborative partnerships between research universities and private industries is essential for advancing Saudi Arabia’s strategic objective of transitioning from an economy dependent on natural resources to one driven by knowledge-based industries. ^41^ These partnerships have facilitated cross-border knowledge exchange and coauthorship of impactful research, aligning with Saudi Arabia’s strategic goal of fostering global academic alliances.

One of the most salient insights from this study is the correlation between multiauthored, collaborative publications and heightened citation and viewership impacts. Multiauthored collaborative publications help increase the visibility of Saudi research on the global stage, contributing to the country’s scientific reputation and influence. ^19^ This finding underscores the value of collective research efforts, as interdisciplinary and cross-institutional collaboration significantly enhances research visibility and influence. Knowledge sharing enhances research productivity and quality, as seen in the increased scholarly publications from academic staff in Saudi universities. ^18^ Saudi researchers exhibit a high collaboration ratio, with 81.1% of publications being multi-authored. ^42^ Moreover, the study identifies the expansion of open-access publishing as a critical driver of this visibility. Saudi Arabia ranks 41st globally in terms of OA publications and has the highest percentage of OA publications among countries worldwide. The last ten years have seen a substantial increase in OA publications from the Kingdom, with a notable peak in citations in 2015. ^32^ Open-access publications have democratized access to scholarly work, facilitating broader dissemination and increasing the readership of Saudi Arabia’s research output.

Saudi Arabia’s economy has heavily relied on oil revenues, which constitute around 45% of its GDP. ^43^ The study’s examination of the relationship between economic indicators and research performance offers a nuanced perspective. The per capita GDP is influenced by various factors such as oil prices, government spending, and trade openness, while Saudi Arabia’s GDP per capita remains lower than that of Western economies such as the United States and several European nations, the analysis reveals no direct association between GDP per capita and research outcomes. This finding indicates that targeted investments in research capacity-building and institutional infrastructure, rather than macroeconomic indicators, are the primary drivers of research success in Saudi Arabia. Saudi Arabia has invested heavily in education and research, with a budget of $56.56 billion in 2014 and numerous universities and research institutes established across the country. ^44^ The study’s findings challenge conventional assumptions that economic wealth alone determines research productivity, thereby highlighting the role of strategic planning and investment.

Despite its comprehensive scope, this study has certain limitations. Addressing these limitations could provide a more nuanced understanding of the underlying factors influencing research productivity and impact. For example, incorporating additional forms of scholarly outputs such as patents, books, and case studies, as well as considering input factors such as research funding, faculty capacity, and institutional support, would offer a more holistic view of Saudi Arabia’s research ecosystem and better inform policy development and strategic planning. It focuses primarily on journal publications indexed in Scopus, excluding other significant scholarly outputs such as patents, books, and case studies. Additionally, input factors such as research funding, faculty capacity, and institutional support—critical elements for evaluating research ecosystems—were not considered in this analysis. The transformation of Saudi Arabia’s research landscape is characterized by substantial growth in productivity, collaboration, and impact, reflecting the nation’s ambition to become a globally influential research hub. Future studies should incorporate these variables to provide a more holistic assessment of Saudi Arabia’s research ecosystem and the factors driving its growth.

## 5. Conclusion

In Summary, this study highlights the significant growth and advancement in Saudi Arabia’s research productivity and impact from 2013 to 2022. Notably, research output has surged by 320%, accompanied by an impressive annual growth rate of 16%, reflecting the nation’s deliberate and strategic efforts to enhance its global research competitiveness. The analysis identifies shifts in research themes, increased collaboration, and improvements in scientific influence. Prominent institutions, such as King Saud University and Prince Sultan University, have demonstrated exceptional capacity in promoting international collaborations and generating impactful research outputs. Additionally, international partnerships, particularly with Egypt, India, and the United States, have quadrupled, underscoring Saudi Arabia’s proactive approach to forming strategic global alliances.

Another critical trend identified is the marked increase in open-access publishing, substantially broadening the international dissemination and accessibility of Saudi research contributions. This growth in open-access publication aligns closely with global trends aimed at democratizing scholarly knowledge and enhancing research visibility. Consequently, Saudi Arabia positions itself prominently within the global movement toward increased openness and transparency in scholarly communication. Furthermore, these findings challenge traditional assumptions linking higher GDP per capita directly to superior research outcomes. Instead, the study emphasizes that targeted investments in research infrastructure, international collaboration, and open-access practices are pivotal to achieving sustained research excellence.

## Ethics and consent

Ethical approval and consent were not required.

## Data Availability

Open Science Framework: Extended data for Saudi bibliometric data are archived at https://doi.org/10.17605/OSF.IO/S9EGC. ^45^ This project contains the following underlying data:
•
Publications_in_Saudi_Arabia 2018-2023.xlsx – Data related to publications from Saudi Arabia during the year 2012-22. Data are available under the terms of the Creative Commons Zero “No rights reserved” data waiver (CC0 1.0 Public domain dedication).
