# Supplementary material for: Comprehensive analysis of Saudi Arabia's research output: a bibliometric study (2013--2022)
Source: F1000Res. 2026 Jan 28;14:61. Originally published 2025 Jan 10. [Version 3] doi: 10.12688/f1000research.160450.3 (PMC12921395; doi:10.12688/f1000research.160450.3)
Supplement: Peer review history

# Reviewer report: Reviewer response for version 3

**DOI:** 10.5256/f1000research.195173.r454810

**Published:** 2026-02-19

**Authors:** Gyanajeet Yumnam

**Affiliations:** 1 Manipur University, Imphal, Manipur, India

The manuscript provides a comprehensive bibliometric assessment of Saudi Arabia’s research output between 2013 and 2022. The study is clearly structured and demonstrates substantial improvement in clarity, methodological transparency, and analytical consistency. The objectives are well defined, and the use of the Scopus database via SciVal is appropriate for conducting a national-level bibliometric evaluation.

 The methodology is adequately described, including the inclusion criteria, the study period, and the statistical procedures. The statistical analyses are correctly applied, and the interpretation of correlation coefficients appropriately distinguishes between the magnitude of association and statistical significance. The presentation of growth trends, collaboration patterns, and citation impact metrics is coherent and supported by well-organized tables and figures.

 Importantly, the availability of underlying data in an open repository strengthens reproducibility and transparency. The discussion contextualizes the findings within broader research policy developments without overstating conclusions. Overall, the study is technically sound, the results support the conclusions, and the manuscript meets acceptable scientific standards for indexing.

Is the work clearly and accurately presented and does it cite the current literature?

Yes

If applicable, is the statistical analysis and its interpretation appropriate?

Yes

Are all the source data underlying the results available to ensure full reproducibility?

Yes

Is the study design appropriate and is the work technically sound?

Yes

Are the conclusions drawn adequately supported by the results?

Yes

Are sufficient details of methods and analysis provided to allow replication by others?

Yes

Reviewer Expertise:

Mycanxpertise includes bibliometric analysis, research evaluation, scientometrics, and quantitative assessment of scholarly publications. I have experience in analyzing citation impact indicators (such as FWCI and collaboration metrics), publication growth trends, and research performance benchmarking at institutional and national levels. I am able to assess the methodological design, statistical analysis, interpretation of bibliometric indicators, and issues related to reproducibility and research transparency presented in this article.

I confirm that I have read this submission and believe that I have an appropriate level of expertise to confirm that it is of an acceptable scientific standard.

# Reviewer report: Reviewer response for version 3

**DOI:** 10.5256/f1000research.195173.r453459

**Published:** 2026-02-03

**Authors:** Yogendra Singh

**Affiliations:** 1 Swami Rama Himalayan University, Dehradun, Uttarakhand, India

I have read the complete article and found that all points raised by me have been taken care of. The article can be indexed without any further revision.

Is the work clearly and accurately presented and does it cite the current literature?

Partly

If applicable, is the statistical analysis and its interpretation appropriate?

Yes

Are all the source data underlying the results available to ensure full reproducibility?

Yes

Is the study design appropriate and is the work technically sound?

Partly

Are the conclusions drawn adequately supported by the results?

Partly

Are sufficient details of methods and analysis provided to allow replication by others?

Yes

Reviewer Expertise:

NA

I confirm that I have read this submission and believe that I have an appropriate level of expertise to confirm that it is of an acceptable scientific standard.

# Reviewer report: Reviewer response for version 2

**DOI:** 10.5256/f1000research.181883.r434135

**Published:** 2026-01-05

**Authors:** Yogendra Singh

**Affiliations:** 1 Swami Rama Himalayan University, Dehradun, Uttarakhand, India

**General Assessment and Summary**

 The manuscript provides a valuable and timely examination of Saudi Arabia's dramatic research growth and its alignment with national strategic goals (Vision 2030). The findings confirm a substantial increase in scholarly output and international collaboration, alongside a rising citation impact (FWCI).

 However, the manuscript suffers from disjointed flow, redundancy, and a lack of critical methodological detail. Specifically, the introduction is overly long and includes excessive literature that is only tangentially related to Saudi Arabia's research productivity.

 The Methods section needs to be clearer about the data collection process and the justification for certain statistical choices.

**1. Introduction Structure and Focus**

 The Introduction is very large and confusing. It starts strongly by establishing the study's goal, but then descends into a lengthy, unstructured literature review.

**2. Methodological Clarity and Justification**

 The Methods section (and Section 1.1) is mixed with the Introduction and needs to be consolidated and clarified.

- *Data Collection* **:** Clarify the exact search string used in SciVal/Scopus. Simply stating "country field tag, Saudi Arabia" is insufficient for reproducibility. Was the affiliation tag, address tag, or country tag used?
- *Timeframe Discrepancy:* The text states the study covers "over a decade (2013–2022)" and "nine-year period." This needs to be consistent. Also, Figures 2 and 3 cover 2018–2023, a period *outside* the stated 2013–2022 window. This discrepancy must be explained and justified, or the study period must be consistently 2013–2022/2023.
- *Statistical Justification* **:** The study uses the Kruskal–Wallis H test and Mann–Whitney U test (non-parametric tests) but describes continuous variables with means and standard deviations (parametric description).

**3. Interpretation of Results and Correlation Analysis**

- *Correlation with GDP* **:** The text states: "we did not find a strong positive correlation between per capita GDP and research outcomes (r = 0.600, p value= 0.067)." However, r=0.600 is conventionally considered a moderate-to-strong positive correlation (as per the authors' own interpretation scale, 0.50–0.74 is strong). The issue is the p-value (0.067), which is *just* above the $0.05 significance threshold.

**Specific Minor Comments**

- *Redundancy* **:** The analysis of the increase in publications (Section 1.2) is highly repetitive. The text states the number of publications increased "significantly by 320% over a period of ten years," then restates the growth rate of 16.0%, and then repeats the highest growth years. Consolidate these details.

 

- *Terminology* **:** Define abbreviations upon first use in the abstract or main text (e.g., FWCI, FWVI, OER, LIS, NCAAA, KPIs) even if they are common in the field.
- *Figures 6a and 6b* **:** Figure 6 is described as two parts ("Figure 6a" and "Figure 6b") but only one is visible/described in detail ("maximum number of international collaborations for each year"). Ensure both parts are presented or described clearly.
- *Table 3 Collaboration Counts* **:** The percentages in Table 3 should be re-checked. Egypt's 17.0% (31,254 papers) of 183,536 is correct. However, India and Pakistan are both listed as 5.0% when one is 10,560 and the other is 9,112. Ensure the percentages accurately reflect the reported document counts for clarity.
- *All the tables and figure should mention the source of data*

Is the work clearly and accurately presented and does it cite the current literature?

Partly

If applicable, is the statistical analysis and its interpretation appropriate?

Yes

Are all the source data underlying the results available to ensure full reproducibility?

Yes

Is the study design appropriate and is the work technically sound?

Partly

Are the conclusions drawn adequately supported by the results?

Partly

Are sufficient details of methods and analysis provided to allow replication by others?

Yes

Reviewer Expertise:

My area of research are bibliometric analysis of biomedical research. I have published sewveral studies in this area

I confirm that I have read this submission and believe that I have an appropriate level of expertise to confirm that it is of an acceptable scientific standard, however I have significant reservations, as outlined above.

# Response

**Published:** 2026-01-12

**Authors:** Santhosh Venkata

**Affiliations:** Manipal Academy of Higher Education, India

**General Assessment and Summary**

 The manuscript provides a valuable and timely examination of Saudi Arabia's dramatic research growth and its alignment with national strategic goals (Vision 2030). The findings confirm a substantial increase in scholarly output and international collaboration, alongside a rising citation impact (FWCI).

 However, the manuscript suffers from disjointed flow, redundancy, and a lack of critical methodological detail. Specifically, the introduction is overly long and includes excessive literature that is only tangentially related to Saudi Arabia's research productivity.

**Response:**

 We sincerely thank the reviewer for the positive overall assessment of the manuscript’s relevance, timeliness, and key findings. In response to the concerns raised, we have substantially revised both the Introduction and the Methodology sections.

 The Methods section needs to be clearer about the data collection process and the justification for certain statistical choices.

**1. Introduction Structure and Focus**

 The Introduction is very large and confusing. It starts strongly by establishing the study's goal, but then descends into a lengthy, unstructured literature review.

**Response:**

 We thank the reviewer for this constructive comment. In response, the Introduction has been substantially restructured and rewritten to improve clarity, coherence, and focus.

**2. Methodological Clarity and Justification**

 The Methods section (and Section 1.1) is mixed with the Introduction and needs to be consolidated and clarified.

- *Data Collection* **:** Clarify the exact search string used in SciVal/Scopus. Simply stating "country field tag, Saudi Arabia" is insufficient for reproducibility. Was the affiliation tag, address tag, or country tag used?

**Response:**

 We thank the reviewer for this important comment. In response, the Methods section has been consolidated and clearly separated from the Introduction, and Section 1.1 has been revised to avoid overlap between methodological description and background content.

  

- *Timeframe Discrepancy:* The text states the study covers "over a decade (2013–2022)" and "nine-year period." This needs to be consistent. Also, Figures 2 and 3 cover 2018–2023, a period *outside* the stated 2013–2022 window. This discrepancy must be explained and justified, or the study period must be consistently 2013–2022/2023.

**Response:**

 We thank the reviewer for identifying this inconsistency. In response, we have carefully reviewed and corrected all references to the study timeframe throughout the manuscript to ensure consistency.

  

- *Statistical Justification* **:** The study uses the Kruskal–Wallis H test and Mann–Whitney U test (non-parametric tests) but describes continuous variables with means and standard deviations (parametric description).

**Response:**

 We thank the reviewer for this important statistical observation. In response, we have revised the manuscript to ensure consistency between descriptive statistics and the statistical tests applied.

 For analyses involving the Kruskal–Wallis H test and Mann–Whitney U test, which are non-parametric methods, continuous variables are now described using median values (and corresponding dispersion measures) in the relevant sections and tables. In addition, mean values were retained only for independent descriptive comparisons, where they are presented purely for summarisation and interpretability purposes and are not used for inferential testing.

**3. Interpretation of Results and Correlation Analysis**

- *Correlation with GDP* **:** The text states: "we did not find a strong positive correlation between per capita GDP and research outcomes (r = 0.600, p value= 0.067)." However, r=0.600 is conventionally considered a moderate-to-strong positive correlation (as per the authors' own interpretation scale, 0.50–0.74 is strong). The issue is the p-value (0.067), which is *just* above the $0.05 significance threshold.

**Response:**

 Thank you for this important observation. We agree with the reviewer that a Pearson correlation coefficient of r = 0.600 indicates a moderate-to-strong positive association based on our stated interpretation scale. To address this issue, we have revised the manuscript to clearly distinguish between the strength of the correlation and its statistical significance.

**Specific Minor Comments**

- *Redundancy* **:** The analysis of the increase in publications (Section 1.2) is highly repetitive. The text states the number of publications increased "significantly by 320% over a period of ten years," then restates the growth rate of 16.0%, and then repeats the highest growth years. Consolidate these details.

**Response:**

 We thank the reviewer for this helpful suggestion. In response, Section 1.2 has been revised to remove redundancy by consolidating all growth-related information into a single, concise narrative.

  

- *Terminology* **:** Define abbreviations upon first use in the abstract or main text (e.g., FWCI, FWVI, OER, LIS, NCAAA, KPIs) even if they are common in the field.

**Response:**

 Thank you for this valuable suggestion. All abbreviations have now been defined at their first occurrence in the abstract and/or main text

  

- *Figures 6a and 6b* **:** Figure 6 is described as two parts ("Figure 6a" and "Figure 6b") but only one is visible/described in detail ("maximum number of international collaborations for each year"). Ensure both parts are presented or described clearly.

**Response:**

 We thank the reviewer for highlighting this issue. In response, both components of Figure 6 are now clearly presented and explicitly described in the manuscript. The figure caption and the corresponding Results text have been revised to clearly distinguish between Figure 6a and Figure 6b, ensuring that both sub-figures are visible, interpreted, and discussed in sufficient detail.

  

- *Table 3 Collaboration Counts* **:** The percentages in Table 3 should be re-checked. Egypt's 17.0% (31,254 papers) of 183,536 is correct. However, India and Pakistan are both listed as 5.0% when one is 10,560 and the other is 9,112. Ensure the percentages accurately reflect the reported document counts for clarity.

**Response:**

 We thank the reviewer for carefully examining the values presented in Table 3. Upon rechecking the calculations, we confirm that the percentages are derived from the total number of collaborative publications (n = 183,536).

 For clarity:

 India: 10,560 / 183,536 = 5.8%

 Pakistan: 9,112 / 183,536 = 5.0%

  

- *All the tables and figure should mention the source of data*

**Response:**

 We thank the reviewer for this suggestion. In response, we have clearly specified the data source in the Methods section, where the origin of all data used in the study is described in detail. As all tables and figures are derived from the same single data source, repeating the source information in each table and figure was avoided to prevent redundancy.

  

- *Is the work clearly and accurately presented, and does it cite the current literature?*

Partly

**Response: **Most of the cited studies are between 2020-2024 and focus on the advancement of research productivity of Saudi Arabia

  

- *Is the study design appropriate and is the work technically sound?*

Partly

**Response: **This is a bibliometric study and is based on the data from large citation and abstract database SCOPUS. The design of this study is similar to previous bibliometric studies

  

- *Are sufficient details of methods and analysis provided to allow replication by others?*

Yes

**Response: **The authors have provided adequate details, and such studies can be replicated

  

- *If applicable, is the statistical analysis and its interpretation appropriate?*

Yes

**Response: **The explanation of statistical analysis has been provided

  

- *Are all the source data underlying the results available to ensure full reproducibility?*

Yes

**Response: **The data is derived from Scopus database and this ensures reproducibility

  

- *Are the conclusions drawn adequately supported by the results?*

Partly

**Response:**

 The conclusion stated has been justified with the help of data compiled and analysed.

# Reviewer report: Reviewer response for version 1

**DOI:** 10.5256/f1000research.176354.r371040

**Published:** 2025-04-25

**Authors:** Mahir Mirah

**Affiliations:** 1 Taibah University, Medina, Al Madinah Province, Saudi Arabia

1. Various citation issues exist in the document. The manuscript contains various unclear expressions such as “Papers 7, 8” and “A study reported in Ref. 10” which need proper citation replacements like “an article by David et al.”

 2. The document uses multiple abbreviations that should be explained when they appear for the first time but currently remain undefined. The set of examples includes UAE as well as HF-HEQ-BI and QA in KSA HEIs and NCAAA standards and KPIs.

 3. Multiple weaknesses appear within the methodology part. The article lacks details about the procedures for selecting studies from a Saudi Arabia within Scopus and how data validity was established and how many researchers performed the search to guarantee reliability and reproducibility. The process documents lack information about how researchers handled the screening of articles.

 4. in figure 4 and figure 2 there are incomplete data for example plasmons;....

 5. The discussion fails to provide sufficient detail regarding the significant results within the research. The manuscript could improve by executing an expanded comparative research method that links its results with other research from the literature base.

 6. The conclusion extends beyond normal boundaries in length. This section should present a succinct overview which contains only the major findings and their essential implications from the study.

 7. The article written or uses artificial intelligence (AI) in the writing process. The manuscript fails to address whether the articles were created by artificial intelligence (AI).

Is the work clearly and accurately presented and does it cite the current literature?

Partly

If applicable, is the statistical analysis and its interpretation appropriate?

Yes

Are all the source data underlying the results available to ensure full reproducibility?

Yes

Is the study design appropriate and is the work technically sound?

Partly

Are the conclusions drawn adequately supported by the results?

Yes

Are sufficient details of methods and analysis provided to allow replication by others?

Yes

Reviewer Expertise:

I have earned both my PhD in Dental Materials along with my Master’s degree in Endodontics from the University of Manchester located in UK. My research interests include: The scientific field of dental sciences utilizes systematic reviews and narrative reviews as part of its research methods. Bibliometric analyses, original studies, and clinical cases. I conduct studies in dental materials through my work with composites and adhesives and restorative technology. The field of endodontics includes the use of instrumentation functions and obturation methods and regenerative treatment procedures. General dentistry research methods and evidence-based dental practice form broader aspects of my study. I possess the ability to perform competent manuscript assessment within the fields of dental materials as well as endodontics and general dentistry-related research.

I confirm that I have read this submission and believe that I have an appropriate level of expertise to state that I do not consider it to be of an acceptable scientific standard, for reasons outlined above.

# Response

**Published:** 2025-05-09

**Authors:** Santhosh Venkata

**Affiliations:** Manipal Academy of Higher Education, India

1. Various citation issues exist in the document. The manuscript contains various unclear expressions such as “Papers 7, 8” and “A study reported in Ref. 10” which need proper citation replacements like “an article by David et al.”

**Response: **As suggested, we have updated

 2. The document uses multiple abbreviations that should be explained when they appear for the first time but currently remain undefined. The set of examples includes UAE as well as HF-HEQ-BI and QA in KSA HEIs and NCAAA standards and KPIs.

**Response: **Suggestions are incorporated

 3. Multiple weaknesses appear within the methodology part. The article lacks details about the procedures for selecting studies from a Saudi Arabia within Scopus and how data validity was established and how many researchers performed the search to guarantee reliability and reproducibility. The process documents lack information about how researchers handled the screening of articles.

**Response: **As per the suggestion, methodology has been updated.

 4. in figure 4 and figure 2 there are incomplete data for example plasmons;....

**Response:  **The chart type has been changed to address the discrepancy.

 5. The discussion fails to provide sufficient detail regarding the significant results within the research. The manuscript could improve by executing an expanded comparative research method that links its results with other research from the literature base.

**Response:  **The discussion part has been updated, and now it talks more about the observations from the research.

 6. The conclusion extends beyond normal boundaries in length. This section should present a succinct overview which contains only the major findings and their essential implications from the study.

**Response:  **The conclusion has been condensed for greater impact and clarity.

 7. The article written or uses artificial intelligence (AI) in the writing process. The manuscript fails to address whether the articles were created by artificial intelligence (AI).

**Response:  **AI tools are not used for writing /editing the present submitted manuscript.
